# Mycotoxins, Phytoestrogens and Other Secondary Metabolites in Austrian Pastures: Occurrences, Contamination Levels and Implications of Geo-Climatic Factors

**DOI:** 10.3390/toxins13070460

**Published:** 2021-06-30

**Authors:** Felipe Penagos-Tabares, Ratchaneewan Khiaosa-ard, Veronika Nagl, Johannes Faas, Timothy Jenkins, Michael Sulyok, Qendrim Zebeli

**Affiliations:** 1 Institute of Animal Nutrition and Functional Plant Compounds, University of Veterinary Medicine, Veterinaerplatz 1, 1210 Vienna, Austria; Felipe.PenagosTabares@vetmeduni.ac.at (F.P.-T.); Qendrim.Zebeli@vetmeduni.ac.at (Q.Z.); 2 BIOMIN Research Center, Technopark 1, 3430 Tulln, Austria; Veronika.Nagl@dsm.com (V.N.); Johannes.Faas@dsm.com (J.F.); timothy.jenkins@bio-ferm.com (T.J.); 3 Department IFA-Tulln, University of Natural Resources and Life Sciences, Vienna (BOKU), Konrad Lorenzstrasse 20, 3430 Tulln, Austria; michael.sulyok@boku.ac.at; 4 Christian-Doppler-Laboratory for Innovative Gut Health Concepts in Livestock (CDL-LiveGUT), Department for Farm Animals and Veterinary Public Health, University of Veterinary Medicine, Veterinaerplatz 1, 1210 Vienna, Austria

**Keywords:** pasture, mycotoxin, fungal metabolite, phytoestrogen, cyanogenic glucoside, ergot alkaloid, temperature, dairy cattle

## Abstract

Pastures are key feed sources for dairy production and can be contaminated with several secondary metabolites from fungi and plants with toxic or endocrine-disrupting activities, which possess a risk for the health, reproduction and performance of cattle. This exploratory study aimed to determine the co-occurrences and concentrations of a wide range of mycotoxins, phytoestrogens and other secondary metabolites in grazing pastures. Representative samples of pastures were collected from 18 Austrian dairy farms (one sample per farm) between April to October 2019. After sample preparation (drying and milling) the pastures were subjected to multi-metabolite analysis using LC-MS/MS. In total, 68 metabolites were detected, including regulated zearalenone and deoxynivalenol (range: 2.16–138 and 107–505 μg/kg on a dry matter (DM) basis, respectively), modified (3-deoxynivalenol-glucoside, HT-2-glucoside) and emerging *Fusarium* mycotoxins (e.g., enniatins), ergot alkaloids and *Alternaria* metabolites along with phytoestrogens and other metabolites. Aflatoxins, fumonisins, T-2 toxin, HT-2 toxin and ochratoxins were not detected. Of the geo-climatic factors and botanical diversity investigated, the environment temperature (average of 2 pre-sampling months and the sampling month) was the most influential factor. The number of fungal metabolites linearly increased with increasing temperatures and temperatures exceeding 15 °C triggered an exponential increment in the concentrations of *Fusarium* and *Alternaria* metabolites and ergot alkaloids. In conclusion, even though the levels of regulated mycotoxins detected were below the EU guidance levels, the long-term exposure along with co-occurrence with modified and emerging mycotoxins might be an underestimated risk for grazing and forage-fed livestock. The one-year preliminary data points out a dominant effect of environmental temperature in the diversity and contamination level of fungal metabolites in pastures.

## 1. Introduction

Grasses and grass-legume mixtures are essential sources of nutrients for herbivores, which can be consumed directly as fresh pastures and preserved as silage and hay. Pastures can be a source of toxic or endocrine-disrupting secondary metabolites originated from some plants, fungi, algae, bacteria and lichens residing in the pasture, which can induce a wide range of animal disorders [1,2,3]. Among these metabolites, mycotoxins, low molecular weight molecules produced by endophytic and epiphytic fungi, are one of the most relevant groups of metabolites due to their high incidence and their negative effects. The contamination of pastures marks an initial point of mycotoxins entering the feed chain. It has been shown that these fungal compounds can represent a risk for animals during grazing and stable periods, causing mycotoxicoses [1,4,5]. Even though ruminants are more resistant to mycotoxins than monogastrics, metabolic and dietary particularities of high producing animals seem to reduce the rumen’s detoxifying ability, thereby increasing the risk of subclinical and clinical health disorders, impairing fertility and affecting productivity [6,7,8].

In general, less information is available regarding mycotoxin levels in pastures compared to the data in grains and conserved feeds [9,10]. Furthermore, although hundreds of compounds have been considered mycotoxins, most studies investigated a limited number of mycotoxins in pastures and other agricultural commodities [11,12]. The most investigated mycotoxins in pastures include the strictly regulated aflatoxin B1 (AFB1) and other mycotoxins with guidance levels (deoxynivalenol (DON), zearalenone (ZEN), fumonisins (FBs), ochratoxin A as well as T-2 and HT-2 toxin) [13,14,15,16,17], which are addressed by the European legislation [18,19]. The ergot sclerotia are also regulated and monitoring of ergot alkaloids (EAs) in food and feed is recommended by the EU [20]. Other relevant but less studied groups of fungal toxins are the modified and emerging mycotoxins. Modified mycotoxins are structurally changed metabolites of the parent forms. These compounds result from biological or chemical modifications. [21]. The emerging mycotoxins have been described as those that are legislatively unregulated and non-regularly analysed, but which occur frequently in agricultural commodities [22]. In addition to single effects, there are toxicological interactions (addition, synergism, potentiation and antagonism) among mycotoxins and other fungal metabolites, which may have implications on animal’s health and reproduction, and this necessitates more research and risk assessment from holistic and integrative approaches [12,23,24]. For instance, synergistic interactions of ZEN, trichothecenes, EAs and other mycotoxins contained in pastures have been discussed as a potential cause of infertility in grazing sheep and cattle [13].

Additionally, pastures are the source of plant secondary compounds such as phytoestrogens (PEs), pyrrolizidine alkaloids, cyanogenic glucosides (CGs), among others, which, at certain dietary levels, may induce detrimental effects on animal health and reproduction [1,25,26,27,28]. Negative effects of PEs on the reproduction of ruminants have been associated with pasture legumes such as clovers (*Trifolium* spp) and lucerne/alfalfa (*Medicago sativa*) [27]. In the context of the reproductive performance of livestock, it seems important that co-occurrences of fungal metabolites and PEs are taken into consideration [29,30].

The production of fungal and plant secondary metabolites is influenced by multiple biological (e.g., species, variety, plant age, parasitic and symbiotic interactions) as well as geo-climatic factors (temperature, relative humidity, rainfall, latitude and altitude) [31,32,33,34]. Some studies on pastures have shown that the geographic location, botanical species and sampling season affect the contamination levels of mycotoxins such as T2-toxin, ZEN and EAs [13,15,16]. Updated data and identification of the most influencing factors could assist in the prediction of contamination as well as the development of strategies for optimal management of forage grasses. The present exploratory study aimed to determine, via an LC-MS/MS-based multi-metabolite method, the presence, co-occurrence and concentrations of mycotoxins, PEs as well as other fungal, bacterial, lichenical and unspecific secondary metabolites in grazing pastures of Austrian dairy farms. Furthermore, potential correlations between the concentrations of the metabolites, and geo-climatic factors of the farms (location, altitude, rainfall, humidity, temperature and time of sampling) were evaluated.

## 2. Results

### 2.1. Occurrence and Concentrations of the Detected Metabolites

#### 2.1.1. Groups of Metabolites 

The occurrence and concentrations (average, SD, median, minimum and maximum, expressed in μg/kg on a DM basis) of individual and grouped metabolites are shown in Table 1. The grouped metabolites were classified according to their main producers including *Alternaria*, *Aspergillus*, *Fusarium*, *Penicillium*, lichen-associated fungi, other (non-identified) fungi and unspecific (i.e., metabolites produced by fungi, bacterial and/or plants), or according to the kind of metabolites (EAs, PEs and CGs) based on previous reports [35,36]. In total 68 out of 481 targeted fungal, plant, lichenical and unspecific metabolites were detected in the studied pastures samples (Supplementary Table S1), consisting of 48 fungal compounds (over 30 known as mycotoxins), 11 plant and 9 unspecific metabolites (Table 1).

**Figure 1:**
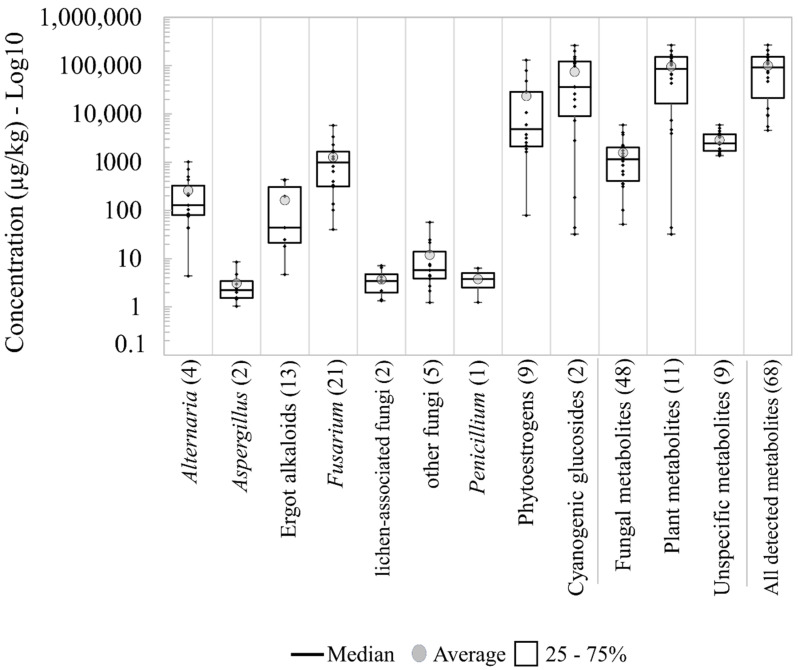
Boxplots for log_10_ concentrations of metabolite groups detected in the pasture samples taken from 18 Austrian dairy farms. The number in parentheses is the number of total detected metabolites per group.

In total, 21 metabolites produced primarily by *Fusarium* spp. were present in the pasture samples and none of the samples was free from *Fusarium* metabolites (Table 1). The number of metabolites derived from *Alternaria* (4), *Aspergillus* (2) and other fungi (5) was considerably smaller with occurrences of 83, 44 and 44 %, respectively. The metabolite group derived from lichen-associated fungi and the EAs occurred in 44 and 39 % of the samples with a total of 2 and 13 metabolites of each respective group were detected. The group of fungal metabolites with the highest average, median and maximum concentrations were produced by *Fusarium*, followed by *Alternaria* and EAs (Figure 1). Only one metabolite produced by *Penicillium* was detected (pestalotin). Metabolites produced by lichen-associated fungi, and other fungal species showed low concentrations with values below 10 μg/kg and 60 μg/kg, respectively (Table 1, Figure 1). 

As shown in Table 1, the groups of plant-derived metabolites, CGs (2 metabolites) and PEs (9 metabolites) were present at high frequencies and high concentrations, with total averages above 70,000 and 20,000 μg/kg, respectively. Nevertheless, the heterogeneity among the samples was evident and many of the samples showed values below the average values (Figure 1). The presence of unspecific metabolites was ubiquitous and more homogenous among the pasture samples, with concentrations between 1370 and 5910 μg/kg. The total concentrations of all metabolites detected ranged from 4560 to 266,700 μg/kg with an average and median around 100,000 μg/kg.

#### 2.1.2. Regulated Mycotoxins and Related Metabolites

**Figure 2:**
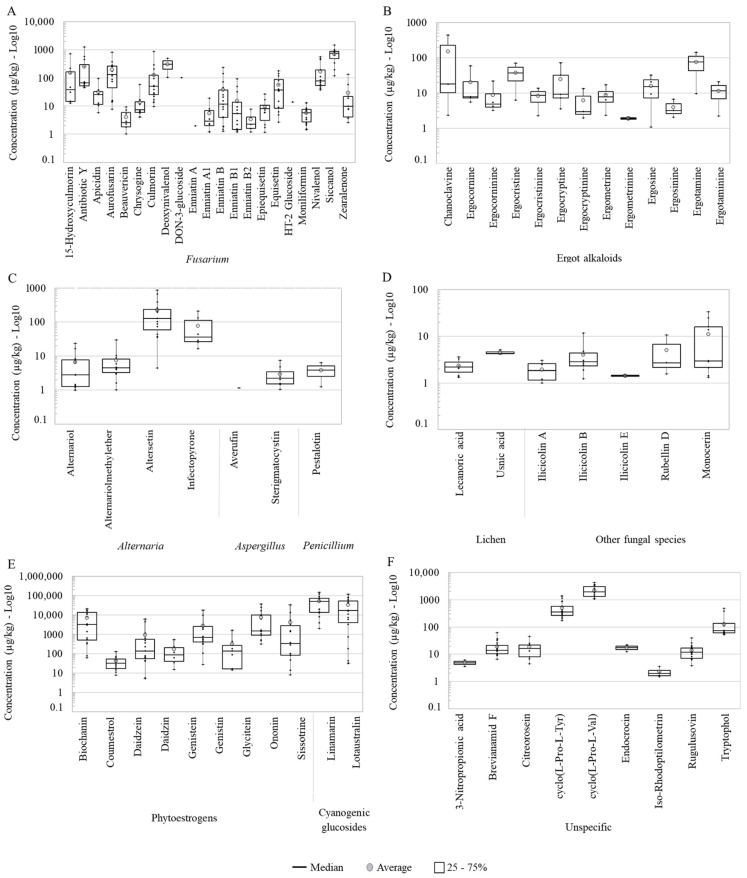
Boxplots for the log_10_ concentrations of individual metabolites in each category: (**A**–**D**) fungal, (**E**) plant and (**F**) unspecific metabolites (produced by fungi, plants and/or bacteria) detected in the pasture samples collected in Austrian dairy farms. The exact mean, SD, median, min and maximum values are shown in Table 1.

The regulated AFB1, along with other AFs, FBs, T-2 toxin and OTA and structurally related forms were not detected in the pasture samples. Two regulated *Fusarium* mycotoxins were found: ZEN (50% positive samples; range: 2.61–138 μg/kg), and DON (11%, range: 107–505 μg/kg) (Table 1), being lower than EU guidance values: 500 and 5000 μg/kg (at 88% DM), respectively. Related to DON, nivalenol (NIV) occurred in more than 80% of the samples with concentrations ranged from 38.1 to 574 μg/kg of the tested pasture samples. The modified mycotoxins DON-3-glucoside (D3G) and HT-2-glucoside (HT-2G) co-occurred in the same sample with concentrations of 102 and 14.0 μg/kg, respectively (Table 1, Figure 2A).

The detected concentrations of individual EAs in the pasture samples ranged from 4.70 to 435 μg/kg (Table 1). In total, 13 different EAs were identified. Chanoclavine and ergotamine showed the superior mean concentrations of the group, 152 and 75.7 μg/kg, in that order. The rest of EAs contained average concentrations below 40 μg/kg. The presence of chanoclavine in the samples was highly heterogeneous, ranging from 2.35–435 μg/kg, but the median of ergotamine was higher than chanoclavine (Table 1, Figure 2B). Other targeted but not detected EAs were agroclavine, dihydroergosine, dihydrolysergol, elymoclavine, epoxyagroclavine, ergine and ergovaline (Supplementary Table S1).

#### 2.1.3. Emerging Mycotoxins

The pasture samples contained 17 compounds considered emerging mycotoxins [37,38,39]. The majority of these emerging mycotoxins were derived from the genera *Fusarium* (in total 14 classified as emerging toxins) and, to a lesser extent, from *Alternaria* (2) and *Aspergillus* (1) (Table 1). Despite the high occurrence of fusarial emerging mycotoxins in the samples, the mean and median concentrations stayed below 150 μg/kg, except for siccanol (758 μg/kg) with noticeable variations among samples (Figure 2A). Concerning the frequency, all samples contained detectable levels of moniliformin. Other frequently found metabolites (over 80% of the pasture samples) were enniatin (ENN) B, ENN B1, culmorin and aurofusarin. Occurring in rates between 50 and 80% of the pasture samples were alternariol (AOH), alternariol methyl ether (AME), epiquisetin, equisetin and siccanol. Siccanol was the *Fusarium* metabolite with the highest average and median concentrations (Figure 2A). Lower occurrences (<50% occurrence) were detected for 15-Hydroxyculmorin, beauvericin (BEA), ENN A1, ENN A and ENN B2, as well as the *Aspergillus*-derived carcinogenic and aflatoxin precursor sterigmatocystin (STC) (Table 1). The concentration of STC showed a higher homogeneity among samples compared to other emerging mycotoxins from *Fusarium* and *Alternaria* (Figure 2A,C).

#### 2.1.4. Other Mycotoxins and Metabolites from Fusarium, Alternaria, Aspergillus, Penicillium and Other Fungi

In addition to the known regulated and emerging mycotoxins, there were many other mycotoxins and metabolites associated with *Fusarium*, *Alternaria, Aspergillus* and *Penicillium* in the pasture samples (Table 1 and Figure 2A,C). Mycotoxin produced by *Fusarium,* including 15-hydroxyculmorin, apicidin, antibiotic Y, aurofusarin and chrysogine had occurrences over 55%, with exception of apicidin (39%) and 15-hydroxyculmorin (44%). Concerning compounds derived from *Alternaria*, altersetin (83%) was the most frequently found metabolite (Table 1). In terms of concentrations, altersetin and infectopyrone were the major detected metabolites produced by *Alternaria* (Figure 2C). The occurrence and concentrations of the *Penicillium* metabolite pestalotin (range: 1.24–6.33 μg/kg) were rather low (Figure 2C).

#### 2.1.5. Metabolites from Lichen-Associated and Other Fungi Genera

The occurrence of metabolites produced by other fungi varied from 11–50% (Table 1). The most frequently found and most produced compound of this group was monocerin (50%; 1.32–33.4 μg/kg). The ilicicolins A, B and E occurred in concentrations below 12 μg/kg. Additionally, two lichen-derived metabolites, lecanoric acid (39%, range: 1.34–3.60 μg/kg) and usnic acid (17%, 4.18–5.10 μg/kg) were detected (Table 1, Figure 2D).

#### 2.1.6. Plant Compounds (Phytoestrogens and Cyanogenic Glycosides) and Unspecific Metabolites

The identified PEs were biochanin, coumestrol, daidzein, genistein, genistin, glycitein, ononin and sissotrine, which occurred in ≥50% of the samples, and the less frequent daidzin (33.3%). Overall, for most PEs levels, the concentrations presented extremely variable, therewith maximum values achieved over 100 times more than the minimum values (Figure 2E). On average, glycitein and biochanin were the PEs that presented levels > 7000 μg/kg and those of genistein and ononin were about 3 times lower. Coumestrol, daidzein, daidzin and genistin had average concentrations below 1000 μg/kg. The CGs, linamarin were the metabolites with the highest concentrations (median, average and maximum) of the study (Table 2, Figure 2E).

Unspecific metabolites are analytes produced by different and unrelated species of fungi, bacteria and/or plants. In this group, five metabolites, namely brevianamide F, cyclo (L-Pro-L-Tyr), cyclo (L-Pro-L-Val), rugulusovine and tryptophol were present in all pasture samples and showed the highest levels of this category. The following unspecific compounds were detected less frequently: citreorosein (50%), iso-rhodoptilometrin (22%), 3-nitropropionic acid (11%) and endocrocin (11%) (Table 2, Figure 2F).

### 2.2. Co-Occurrence of Mycotoxins and Other Metabolites

**Figure 3:**
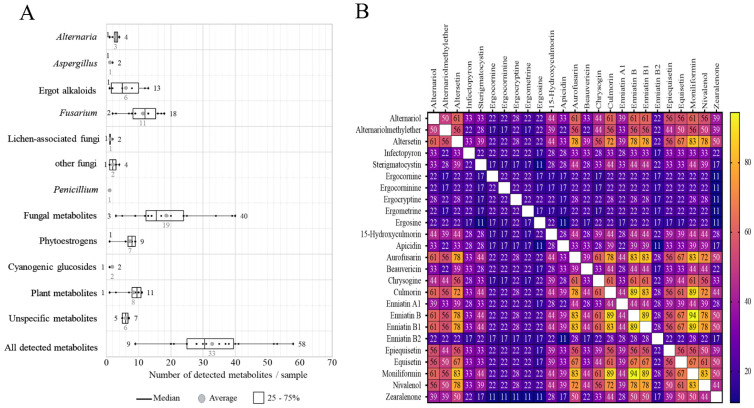
Co-occurrences of mycotoxins and other secondary metabolites detected in the pasture samples taken from 18 Austrian dairy farms. (**A**) Boxplots showing the number of metabolites per sample in each metabolite group. (**B**) Heatmap indicating the co-occurrence of the major mycotoxins (i.e., which occurred ≥20% of total samples) detected in the pastures.

The number of detected metabolites per sample are shown in Figure 3A. On average, 33 (range: 9–58) metabolites per sample were found and on average 7 PEs were detected per sample. On average 19 fungal metabolites (range: 3–40) were present in a sample. All pasture samples contained at least one CG. 

The co-occurrence analyses of mycotoxins and metabolites are shown in Figure 3B. 94% of the pasture samples contained 20 or more metabolites. The most frequent combinations of mycotoxins detected in the pasture samples were MON and ENN B (94%), ENN B and ENN B1 (89%), CUL and ENN B (89%), aurofusarin and ENN B (83%) and aurofusarin and MON (83%), all of which are *Fusarium* metabolites. The combination of the other *Fusarium* metabolites ZEN and NIV was found in 44% of the samples. Interestingly, most of the samples showed co-occurrences between *Fusarium* and *Alternaria* metabolites, especially for altersetin, which co-occurred with several *Fusarium* emerging mycotoxins (aurofusarin, CUL, ENN B and MON) in more than 70% of the samples and with ZEN in 50% of the samples. Two mycoestrogens from *Alternaria*, AOH and AME, had co-occurrences of 39% with ZEN. Up to 30% of the tested pastures showed co-contamination between detected EAs and *Fusarium* mycotoxins (Figure 3B).

### 2.3. Effect of Season, Locations and Pasture Diversity

Sampling was carried out once per farm during the grazing season of the year 2019. Subsequently, the sampling season was classified as early (April–June) and late (August–October). There was a significant difference in the co-contamination of metabolites (i.e., the number of metabolites/sample) and concentrations of several groups of mycotoxins and metabolites between the sampling seasons (Table 2). Samples collected late had higher levels of co-contamination of fungal metabolites (*p* = 0.012) and number of total metabolites increased (*p* = 0.008) compared to those of early sampling. A similar trend occurred with the concentrations of total metabolites from total fungi (*p* = 0.005), *Fusarium* (*p* = 0.041) and other fungal species (*p* = 0.041), which resulted in higher concentrations in the pastures during the late sampling season than in the early sampling. The location (classified by their federal state) and the pasture diversity did not affect the co-occurrence or the levels of metabolite groups in the tested pasture samples (data not shown).

**Figure 4:**
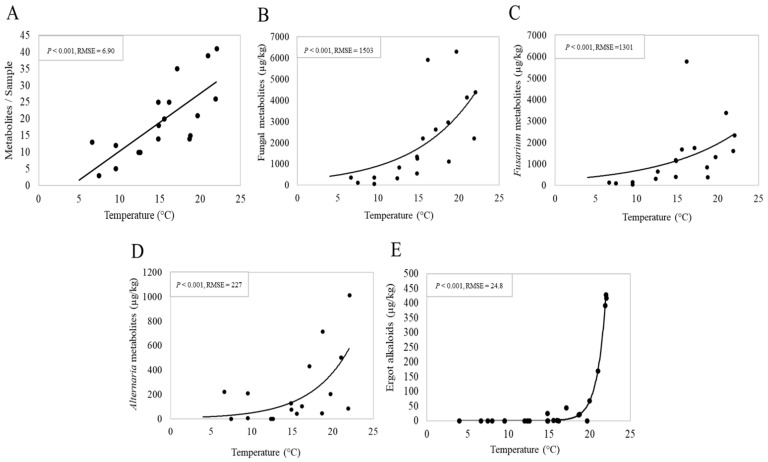
Linear regression showing a relationship between 3-month average temperature (the mean of 2 months pre-sampling and the sampling month) and the number of metabolites per sample (**A**) or concentrations of total and individual group of fungal metabolites (**B**–**E**). RMSE: Root mean square error.

We examined the influence of altitude and the climatic variables (temperature, humidity and rainfall at different time scales including whole-year average, 3-month average and sampling-month average). In line with the season effect, among the variables investigated, the 3-month average temperature (the mean of 2 months pre-sampling and the sampling month) was the only climatic variable that showed a significant correlation with the mycotoxin data (detailed data not shown). As shown in Figure 4A, the 3-month average temperature showed a significant positive relationship (*p* < 0.001) with the co-occurrence of metabolites. Specifically, the number of metabolites per sample linearly increased with increasing temperature. Regression suggests an increase of 2.06 ± 0.5 fungal metabolites/sample per one degree Celsius of the 3-month average temperature (*p* < 0.001). Concentrations of total fungal metabolites along with *Fusarium* metabolites, EAs and *Alternaria* metabolites showed an exponential increment in response to the 3-month temperature. Accordingly, the concentrations of *Fusarium*, *Alternaria* and total fungal metabolites in the pasture samples remained comparably low when the temperature was below 15 °C and rapidly rose thereafter as underlined by a higher slope after this critical temperature (Figure 4B–D). Interestingly, the EAs concentrations were very low (<70 μg/kg) or absent at the temperature below 20 °C and rose strongly to concentrations over 400 μg/kg at 22 °C (Figure 4E).

## 3. Discussion

Mycotoxin contamination is an important feed safety issue that also attributes to the food safety issue due to the transfer of certain mycotoxins to animal products. Most of the previous studies have focused mostly on AFs, EAs, as well as *Fusarium* toxins DON, ZEN, T-2 toxin, HT-2 toxin and FBs [13,14,15,16,17]. There is a growing concern about the presence of modified and emerging mycotoxins in diets and associated risks for human and animal health according to the European Food Safety Authority (EFSA). Scientific opinions of EFSA and other authors have underlined the need for new information concerning the (co-) occurrence of those groups of fungal metabolites in foods and feed along with toxicity data [12,40,41,42,43,44]. To the best of our knowledge, the present study is the first study in Europe that documented the occurrences not only of mycotoxins but also of some relevant plant-derived compounds (phytoestrogens and cyanogenic glycosides) as well as unspecific metabolites in pastures used for dairy production, which underlines pasture as a potential route of mycotoxins and other metabolites entering the feed chain.

The high occurrence of *Fusarium* metabolites found in the present study coincided with the findings of Nichea et al. in pastures collected in Argentina [45,46]. This corroborates once again the status of *Fusarium* as one of the most widespread fungal species in crops-growing areas of the planet and as a significant contributor to mycotoxin contamination in animal feeds [47,48,49]. Several *Fusarium* spp. capable of producing the mycoestrogen ZEN are common in pasture microflora [50], which explains the considerable incidence (50%) of this mycoestrogen in the Austrian pastures observed in the present study. Nevertheless, the detected levels of ZEN were below the guidance level (500 μg/kg DM) in feed intended for dairy cows recommended by the European Commission [19] and were low in comparison with previous studies from other geographic regions including New Zealand (max: ~4000 μg/kg) [50], Australia (36%, max: 3006 μg/kg DM) [13], Argentina (90% in 2011 and 81% in 2014, max: 2120 μg/kg), United States (61%, max: 1936 μg/kg) [51] and Russia (up to 5750 μg/kg) [16]. Studies on the effects of feeding ZEN contaminated oats at a concentration of 1.25 mg ZEN/kg feed DM were evaluated in heifers by EFSA (2004) revealing no related impacts on the oestrus cycle or histological structure of reproductive organs [52]. A study showed that ZEN intakes greater than 3 mg/ewe/day adversely affected reproduction, depressing ovulation rated and lambing percentages [53]. Based on these previous reports, by assuming an approximate 20 kg DM intake of pastures, found levels of ZEN in the Austrian pastures would not represent a high risk for ZEN-associated fertility problems in dairy cows. However, previous studies have not considered a synergistic effect related to co-occurrences of ZEN with other mycotoxins and xenoestrogens such PEs, which seems plausible [13].

Another important *Fusarium* mycotoxin is the type B trichothecene DON, which was found in a low frequency (11%) with a maximum concentration of 505 μg/kg being lower than the European guidance level (5000 μg/kg DM) [19]. Our findings stayed within the concentration range found in an Australian survey (129–682 µg/kg DM), although the authors reported DON at a higher frequency of 46% [13]. The maximum level of DON reported by Štýbnarová (2016) in Czech pastures was 715 μg/kg DM [54]. Remarkably, another type-B trichothecene NIV was detected at a much greater frequency (83%) with maximum concentrations of 574 µg/kg DM. Notably, an in vivo study using a mice model indicated that NIV has a higher oral toxic capacity (lower LD_50_) than DON [55]. Due to its structural and toxicological similarities to DON, NIV has exhibited synergistic interactions in co-occurrence with DON and other types B trichothecenes in cell culture models [56,57,58]. Interestingly, another study found antagonistic effects [59]. The risks related to long-term exposure to low levels of NIV in animal feed are challenging to assess due to the limited information available in livestock species [40]. The emerging *Fusarium* mycotoxin ENN B was one of the most prevalent mycotoxins in the present study (94% occurrence), which was higher compared to a report in Argentinean grasses (70% occurrence) [45]. Metabolism of ENNs and BEA has been examined in monogastric animals, while data in ruminants are limited [60]. It is known that these compounds have antifungal, antibiotic and cytotoxic properties [61]. Our and other studies have underlined the significance of non-regulated (emerging) mycotoxins due to their high frequency. The impact of these emerging mycotoxins on dairy cattle as well as their influence on the rumen microbial ecology and digestive physiology have yet to be addressed [38]. 

Ergot alkaloids are produced mostly by *Claviceps* and *Epichloë* spp. These fungal species are known to parasitize a wide spectrum of monocotyledonous plants of different taxonomical families such as *Poaceae*, which includes forage grasses and cereals [62,63,64]. Ingestion of EAs by livestock can trigger a range of impacts from decreased performance and reduced fertility to acute clinical signs of ergotism including hyperthermia, convulsions, gangrene of the extremities and death [65,66,67]. Ergotism is primarily associated with *Claviceps* toxin ergotamine, which was detected in our samples with a greater mean concentration than most of the EAs detected. Fescue toxicosis is linked to ergovaline, produced by *Neotyphodium coenophialum* in fescue grass (*Festuca arundinacea*) [65]. Ergovaline has been reported as the causal agent of severe intoxications in dairy farms [68,69]. These compounds can induce various cardiovascular, neurological as well as endocrinal effects [70,71,72]. Ergovaline was, however, not detected in the present study probably because only 2 pasture samples contained *Festuca pratensis* and it was a minor species in the pasture in both cases (Supplementary Table S2). Subclinical estrogenism has been proved as a significant disruptor of the reproductive performance of small ruminant herds in both Australia [73] and New Zealand [74]. It was proposed that feed contaminated with 250 μg/kg of EAs should not be fed to pregnant or lactating animals due to a higher risk of abortion and agalactia syndrome [75]. Two of the seven Austrian pastures contaminated with EAs contained a total concentration (418 and 434 μg/kg DM) above this recommendation, underlining a potential risk of pastures due to possibilities for high burdens of EAs. This emphasizes the need for close surveillance of EA contamination in pastures. 

Concerning *Aspergillus* derived metabolites, although AFs were not detected, averufin and STC, two of their precursors were detected in our pasture samples [76,77]. Sterigmatocystin itself is known as a carcinogenic compound with high toxicological relevance. In general, the information available on exposure data of dairy cows to these precursors of AF is scarce [41]. Two detected emerging *Alternaria* mycotoxins, AOH and AME, belong to the chemical groups dibenzo-α-pyrones, are toxicologically relevant [78] and considered mycoestrogens, showing strong synergistic estrogenic effects in combination with the fusarial mycoestrogen ZEN even at very low concentrations [79]. However, EFSA declared that research data and information are scarce regarding toxic effects of *Alternaria* toxins on farm animals and companion animals and their occurrence in the feed, thus the health risk for different species associated with *Alternaria* toxins in feeds are not known [80]. The most occurrent toxin from *Alternaria* in this study was ALS with a mean concentration of 220 µg/kg DM but the maximum concentration reached 861 µg/kg DM. This toxin generated by species from the genus *Alternaria* has antimicrobial activity against several bacteria [81]. We also observed the co-occurrence of *Alternaria* mycotoxins with emerging *Fusarium* mycotoxins (such as ENNs and BEA, also with bactericidal properties) [60], thus ingestion of contaminated feed may have consequences for the ruminal bacterial community and functions that are important for the health and productivity of a ruminant. 

Interestingly, we observed that the concentrations of both *Fusarium* and *Alternaria* metabolites responded to increasing temperature in a similar pattern with a critical temperature of 15 °C triggering the exponential increment of these metabolites. This matches with the fact that temperature is a primary determining factor implicated in the modulation of fungal growth and the subsequent mycotoxin production [82,83]. The effect on selective groups of fungal metabolites may suggest that the metabolism of these fungi driven by temperature may be interconnected. Fuchs et al., (2017) projected that the endophyte-mediated intoxications in livestock may increase on European grasslands with global warming [84]. The findings of the temperature effect reinforce the idea that global warming contributes to mycotoxin risk on crops [85,86,87]. Nevertheless, due to the small sample size, variations among the farms and short time of observation, the results presented in this exploratory study should be regarded as preliminary findings and thus must be interpreted with caution. Our results also suggest that the number of fungal metabolites was higher in pastures sampled later in the grazing season (July and October), which should be confirmed by future studies. Furthermore, the production of fungal secondary metabolites is mediated by several biotic and abiotic factors, [82], which cannot be entirely covered by the present study. Therefore, future studies with a larger sample size, more geographic locations and extended years of observation are pivotal to verify the current results regarding the critical temperature and its association with other geo-climatic and botanical factors for elevating mycotoxin contamination of pastures.

Phytoestrogens are produced, among other kinds of plants, by legumes such as *Trifolium prantense*, *T. repens* and *M. sativa*. [27]. The detected PEs in the present study belong to two different categories: isoflavones (biochanin A, daidzein, daidzin, glycitein, genistein, genistin, onionine and sissotrine) and coumestans (coumestrol) [88,89]. The latter category seems to be more potent in inducing infertility problems [27], considering that coumestrol has a superior affinity to the 17β-estradiol than the isoflavone-derived equol [90]. Coumestrol can induce an acute or sub-acute decline of reproductive efficiency in sheep, cattle and horses [91,92,93]. The critical range of coumestrol in cattle feed was reported to be around 18–180 mg/kg [88]. In the current study, isoflavones were the predominant kind of PE and were detected in low quantities (7.9–129 μg/kg DM). Still, the impact of relatively low coumestan concentrations should not be ignored if the diet contains other xenoestrogens (e.g., isoflavones and mycoestrogens) [79], which were also present in the examined samples. Our results also underlined the co-occurrence of phytoestrogens and the mycoestrogen ZEN in pastures. Considering the estrogenic nature of both kind of compounds, an additive/synergistic interaction has been suggested [23]. Given the possibilities for synergistic effects of combinations of toxins, endocrine disruptors and other metabolites, these complex mixtures naturally occurring in pastures might be an underestimated risk for the health and productivity of dairy cattle, especially for high-producing cows with high feed intake.

## 4. Conclusions

The present study reveals that a broad range of mycotoxins, phytoestrogens and secondary metabolites are detected in pastures grown for dairy farming in Austria. Even though concentrations of individual fungal toxins and metabolites were generally low (often less than 200 μg/kg DM), the total fungal metabolite concentration could reach over 6000 μg/kg DM in pastures. Our data underline *Fusarium* as the major fungi in pastures. Still, the attention should also be paid to possibilities for high burdens of EAs and *Alternaria* mycotoxins in pastures. The preliminary data presented here suggests that an increment in the environmental temperature could drive the increased level of contamination from *Fusarium*, *Alternaria* and EAs in pastures. However, it should be further corroborated considering multifactorial influences from geo-climatic and botanical factors as well as year variations. 

## 5. Materials and Methods

### 5.1. Sampling of Pastures

**Figure 5:**
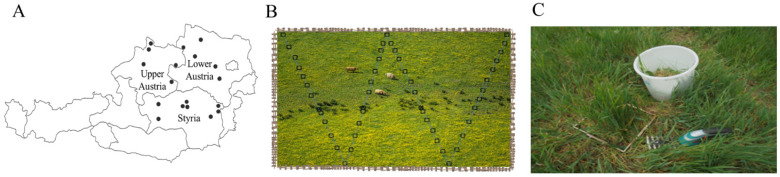
A representative sampling of pastures intended for multi-metabolite analysis. (**A**) Locations of the selected dairy farms (n = 18) in 3 Austrian federal states: Lower Austria, Upper Austria and Styria. (**B**) The sampling pattern (at least 30 incremental samples in a W shape) across a paddock that was being currently grazed at the time of sampling. Sample amount: ≥1–1.5 kg. (**C**) A quadrate (25 cm × 25 cm) used for sampling each incremental sample.

This study was part of a larger project surveying 100 dairy farms in the 3 states leading the country’s dairy production (Lower and Upper Austria along with Styria) for detection of mycotoxins and implications for dairy performances. Of these 100 farms, 18 farms included partial grazing systems for the dairy cows and were selected for this study (Figure 5A). Under informed consent of the farmers, one representative sample of pasture was collected at a one-time point in each farm during the grazing season of 2019 (April–October). In this case, 8 farms were collected in April–June 2019 and 10 farms in August–October 2019. To obtain the representative sample of each farm 30 increment samples (Figure 5B) from a paddock being currently grazed were collected. Each incremental sample was taken from the area of 25 cm × 25 cm of pasture delimited by a metal frame. The pastures were cut 2–3 cm above the soil level using electric grass shears (Figure 5C). The 30 incremental samples were then composited, thoroughly mixed and approximately 1 kg of sample was taken, vacuum packed (−0.7 psi) and stored at −20 °C until sample preparation and analysis. The major botanical species of each sampled paddock were identified based on the morphological features of dissected specimens preserved in a herbarium by an expert. As identified, the sampled pastures contained mixtures of Gramineae (Family: Poaceae, including *Lolium perenne*, *Dactylis glomerata*, *Poa pratensis*, *Festuca pratensis*, *Alopecurus pratensis* and *Phleum pretense*) and Leguminosae (Family: Fabaceae; *Trifolium pretense*, *T. repens* and *Medicago sativa*). Visually, *Gramineae* were the dominating species of all pasture samples, but the exact proportions of individual species were not determined. 

The climatic data (monthly averages of air temperature, air relative humidity and rainfall) of 2019 of the municipalities or districts were collected from the website of the Austrian Agency of Meteorology and Geodynamics (Zentralanstalt für Meteorologie und Geodynamik-ZAMG, https://www.zamg.ac.at/cms/de/klima/klimauebersichten/jahrbuch). The pilot farms were in altitude ranges between 235–1340 m.a.s.l. The annual average temperature values in the areas of the farms ranged from 8.4 to 11.5 °C and the mean annual rainfall was between 502 to 954 mm, concentrated mostly during spring and summer. The average values of relative air humidity of the different locations during 2019 varied between 71.5 and 80%. Climatic data including temperature, humidity and rainfall (annual, monthly and 3-months averages) were checked and recorded for the correlation and regression analyses.

### 5.2. Mycotoxin Analysis

#### 5.2.1. Chemicals and Reagents

Analytical grade reagents and chemicals were used for analysis. Glacial acetic acid (p.a.) and methanol (LC gradient grade) were acquired from Merck (Darmstadt, Germany); ammonium acetate (MS grade) from Sigma-Aldrich (Vienna, Austria) and acetonitrile (LC gradient grade) from VWR International (Leuven, Belgium). Standards of fungal, bacterial, plant and unspecific metabolites were acquired either via donation from various research institutions or purchased from commercial suppliers such as Romer Labs^®^ Inc. (Tulln, Austria), Sigma-Aldrich (Vienna, Austria), Iris Biotech GmbH (Marktredwitz, Germany), Axxora Europe (Lausanne, Switzerland), LGC Promochem GmbH (Wesel, Germany), AnalytiCon Discovery (Potsdam, Germany), Enzo Life Sciences (Lausen, Switzerland), BioAustralis (Smithfield, Australia) and Toronto Research Chemicals (Toronto, Canada). Water was purified successively by reverse osmosis and an Elga Purelab ultra-analytic system from Veolia Water (High Wycombe, UK) to 18.2 MΩ. Stock solutions of each analyte were prepared by dissolving the solid substance, preferably at 250 μg/mL in acetonitrile, but depending on the respective solubility, a few compounds were dissolved in acetonitrile/water 1:1 (*v*/*v*), methanol or water instead as reported by Sulyok et al. [94]. Thirty-four combined working solutions were prepared to precede the spiking experiments by mixing the stock solutions of the corresponding analyte All solutions were stored at −20 °C and allowed to reach room temperature before the analysis.

#### 5.2.2. Sample Preparation, Extraction and Estimation of Apparent Recoveries

The frozen pasture samples were thawed at room temperature for 24 h, then they were air-dried at 65 °C for 48 h. The average DM content of pasture samples was 22.3 ± 8.2% (range: 14.2–35.6%). The dried samples were sequentially milled to a final particle size of ≤0.5 mm. Firstly, the air-dried samples were processed using the cutting mill (SM 300, Retsch GmbH, Haan, Germany) at 1500 rpm for approximately 1 min. The remnant (mostly hard fragments of seeds) was subsequently milled using an ultra-centrifugal mill (ZM 200, Retsch GmbH, Haan, Germany) at 10,000 rpm for approximately 30 s. All milled fractions were combined and homogeneously mixed into one representative sample per farm.

Five grams (±0.01 g) of each homogenized sample were weighed into 50-mL polypropylene conical tubes (Sarstedt, Nümbrecht, Germany) and 20 mL of the extraction solvent (acetonitrile/water/acetic acid 79:20:1, *v*/*v*/*v*) was added. The samples were extracted on a GFL 3017 rotary shaker (GFL, Burgwedel, Germany) in a horizontal position at 180 rpm for 90 min. Then, the tubes were put in a vertical position for 10–15 min for sedimentation. A supernatant of 500 μL of the raw extract was diluted 1:1 with a dilution solvent (acetonitrile/water/acetic acid 20:79:1, *v*/*v*/*v*) in autosampler vials. The injection of 5 μL of the diluted raw extracts into the LC-MS/MS instrument was performed as described by Sulyok et al. 2020 [94]. Quantification was performed from external calibration by serial dilutions of a stock solution of multiple analytes. The results were corrected for apparent recoveries determined through spiking experiments [95].

#### 5.2.3. LC-MS/MS Parameters

The chromatographic method and chromatographic and mass spectrometric parameters used in the current research were carried out at the Department of Agrobiotechnology (IFA-Tulln) at the University of Natural Resources and Life Sciences Vienna (BOKU) in Tulln, Austria and have been described detailed previously [94,95]. This fully validated method enables the accurate quantification of more than 500 fungal, bacterial, plant, lichenical and unspecific secondary metabolites, including all relevant mycotoxins. Analysis was performed with an Agilent 1290 Series HPLC System (Agilent, Waldbronn, Germany) coupled with a QTrap 5500 equipped with a TurboIonSpray electrospray ionization (ESI) source (Sciex, Foster City, CA, USA). Chromatographic separation was performed at 25 °C on a Gemini^®^ C18-column, 150 × 4.6 mm inner diameter, 5 μm particle size, protected by a C18 security guard cartridge, 4 × 3 mm inner diameter (Phenomenex, Torrance, CA, USA). A methanol/water gradient containing 5 mmol/L ammonium acetate and 1% acetic acid was used at 1 mL/min. 

Electrospray ionization-MS/MS was performed in the time-scheduled multiple reaction monitoring (MRM) mode both in positive and negative polarities in two separate chromatographic runs per sample by scanning two fragmentation reactions per analyte. Qualitative and quantitative analyses were performed using Analyst, version 1.5 (AB Sciex, Framingham, MA, USA) and MultiQuant, version 2.0.2 (AB Sciex). The analyte identification was confirmed by the acquisition of two MRMs per analyte, yielding 4.0 identification points according to Commission Decision 2002/657/EC [18]. Furthermore, the LC retention time and the intensity ratio of the two MRM transitions agreed with the related values of an authentic standard within 0.1 min and 30% relative abundance, respectively. Quantification was based on external calibration (linear, 1/x weighted) using a serial dilution of a multi-analyte working solution. Results were corrected using apparent recoveries obtained through spiking experiments. The accuracy of the method is continuously validated by participation in a proficiency testing scheme organized by BIPEA (Gennevilliers, France) with a current rate of z-scores between −2 and 2 of >95% (>1500 results submitted).

### 5.3. Statistical Analysis

Descriptive statistics (occurrences and concentration values: average, median, minimum and maximum) were computed using only the positive values (x ≥ limit of detection (LOD)). Data below LOD were deemed not detectable. Metabolite concentrations below the respective limit of quantification (LOQ) were calculated as LOQ/2. The concentrations are presented on a DM basis in μg/kg–parts per billion (ppb) and on a logarithmic scale (Log10) where applicable. The co-occurrence analysis was performed constructing a matrix with the detection frequencies of the mycotoxins occurring ≥ 20% using Microsoft Excel and the heat map was elaborated by GraphPad Prism (Prism version 9.1, GraphPad Software, San Diego, CA, USA).

For correlations and climatic factors, the statistical analyses were performed using SAS (version 9.4; SAS Institute Inc., Cary, NC, USA). A two-tailed Pearson correlation was accomplished (data not shown) to screen possible significant relationships between the concentrations of the different groups of metabolites and climatic data, followed by the graphical evaluation. Subsequently, targeted pairs were evaluated in detail to quantify their responses. Linear regressions of the 3-month average temperature and the number of fungal metabolites per sample was performed using the Mixed procedure of SAS. The random effect of the farm was considered in the model. For the grouped fungal metabolites showing a non-linear relationship, then the NLIN procedure of SAS was used. An effect of sampling time, farm location or botanical diversity on the concentrations of grouped metabolites was evaluated using the MIXED procedure of SAS. The sampling time was grouped as early (sampled in April to June 2019, n = 8) or late (sampled in August–October 2019, n = 10). The farm location was designated to their federal state including Lower Austria (n = 5), Upper Austria (n = 5) and Styria (n = 8). Two groups of pasture diversity were defined including i) not diverse when one or two botanical species were identified in the samples (n = 11) and ii) diverse when three or more botanical species were detected (n = 7). The statistical model of each geo-climatic factor included a fixed effect of the test factor and a random effect of the farm. The resulting data reported are the least-squares means and standard error of the least-squares mean (SEM).

## Figures and Tables

**Table 1:** Occurrence and concentration of mycotoxins, fungal metabolites, phytoestrogens and other secondary metabolites detected in pastures collected from Austrian dairy farms.

Group	Metabolite	Positive Samples (%) ^1^	Concentration (μg/kg DM) ^2^		
			Average ± SD	Median	Range
*Alternaria*	Alternariol ^3^	61	6.41 ± 7.43	2.81	1.00–23.7
	Alternariolmethylether ^3^	56	7.30 ± 8.30	4.45	1.01–29.4
	Altersetin	83	220 ± 246	127	4.36–861
	Infectopyrone	33	76.5 ± 78.7	36.3	16.3–212
	Total ^4^	83	260 ± 286	128	4.36–1010
*Aspergillus*	Averufin	6	-	-	1.15
	Sterigmatocystin ^3^	44	2.94 ± 2.13	2.21	1.03–7.34
	Total ^4^	44	3.08 ± 2.48	2.21	1.03–8.49
Ergot alkaloids ^5^	Chanoclavine	17	152 ± 245	17.93	2.35–435
	Ergocornine	22	20.1 ± 26.1	7.83	5.57–59.2
	Ergocorninine	22	8.72 ± 8.83	4.86	3.27–21.9
	Ergocristine	17	38.0 ± 31.9	37.5	6.33–70.1
	Ergocristinine	17	8.21 ± 5.71	8.64	2.30–13.7
	Ergocryptine	28	24.8 ± 28.4	9.27	3.6–71.5
	Ergocryptinine	17	6.12 ± 6.30	3.01	1.97–13.4
	Ergometrine	22	8.76 ± 6.19	7.80	2.38–17.1
	Ergometrinine	11	1.92 ± 0.26	1.92	1.73–2.1
	Ergosine	22	15.9 ± 13.5	15.1	1.1–32.1
	Ergosinine	17	3.99 ± 2.39	3.24	2.06–6.66
	Ergotamine	11	75.7 ± 93.3	75.7	9.7–142
	Ergotaminine	11	11.6 ± 13.2	11.6	2.24–20.9
	Total ^4^	39	163 ± 191	43.9	4.70–435
*Fusarium*	15-Hydroxyculmorin ^3^	44	152 ± 243	39.2	13.0–721
	Antibiotic Y	67	254 ± 374	66.5	45.5–1290
	Apicidin ^3^	39	31.3 ± 31.5	25.9	5.84–97.9
	Aurofusarin ^3^	83	196 ± 213	133	7.89–835
	Beauvericin ^3^	44	3.99 ± 3.03	2.6	1.02–9.34
	Chrysogine	61	13.6 ± 15.5	7.42	4.07–58.2
	Culmorin ^3^	89	129 ± 216	51.1	9.53–882
	Deoxynivalenol ^5^	11	306 ± 281	306	107–505
	DON-3-glucoside ^6^	6	-	-	102
	Enniatin A ^3^	6	-	-	2.01
	Enniatin A1 ^3^	44	5.54 ± 6.03	2.92	1.22–19.1
	Enniatin B ^3^	94	38.3 ± 63.9	11.8	1.30–241
	Enniatin B1 ^3^	89	15.3 ± 24.8	5.49	1.19–93.3
	Enniatin B2 ^3^	28	3.41 ± 2.74	2.27	1.19–7.90
	Epiequisetin ^3^	56	9.27 ± 7.96	8.09	1.18–27.2
	Equisetin ^3^	67	57.9 ± 60.4	37.6	2.72–179
	HT-2 Glucoside ^6^	6	-	-	14.0
	Moniliformin ^3^	100	5.70 ± 3.52	5.79	1.45–13.1
	Nivalenol	83	170 ± 182	78.6	38.1–574
	Siccanol ^3^	61	716 ± 392	758	119.3–1480
	Zearalenone ^5^	50	29.6 ± 44.3	9.93	2.61–138
	Sum of enniatins	94	57.4 ± 95.5	18.5	1.3–364
	Sum of type B Trichothecenes	83	218 ± 289	78.6	38.1–1070
	Total ^4^	100	1280 ± 1430	983	40.2–5770
*Penicillium*	Pestalotin	11	3.79 ± 3.60	3.79	1.24–6.33
	Total ^4^	11	3.79 ± 3.60	3.79	1.24–6.33
lichen-associated fungi	Lecanoric acid	39	2.31 ± 0.86	2.17	1.34–3.60
	Usnic acid	17	4.49 ± 0.53	4.19	4.18–5.10
	Total ^4^	44	3.71 ± 2.18	3.44	1.34–7.13
other fungi	Ilicicolin A	22	1.92 ± 0.98	1.83	1.00–3.02
	Ilicicolin B	44	4.00 ± 3.33	2.85	1.23–11.7
	Ilicicolin E	11	1.44 ± 0.11	1.44	1.36–1.51
	Rubellin D	17	5.00 ± 5.00	2.7	1.56–10.7
	Monocerin	50	11.0 ± 11.8	2.97	1.32–33.4
	Total ^4^	72	12.0 ± 15.4	5.73	1.23–56.9
	Sum of fungal metabolites	100	1570 ± 1580	1145	51.7–5880
Phytoestrogens	Biochanin	89	7060 ± 7560	3240	62.1–20,650
	Coumestrol	67	41.6 ± 34.4	32.9	7.88–130
	Daidzein	83	936 ± 1840	139	5.16–6110
	Daidzin	33	167 ± 200	88.7	15.8–543
	Genistein	83	2760 ± 4780	704	28.4–17,550
	Genistin	50	311 ± 513	139	14.6–1630
	Glycitein	83	7470 ± 10,700	1500	315–35,850
	Ononin	83	2230 ± 4210	186	47.1–15,130
	Sissotrine	78	4210 ± 9050	331	8.19–33,070
	Total ^4^	89	23,570 ± 35,920	4850	78.8–130,530
Cyanogenic glucosides	Linamarin	83	50,620 ± 44,880	49,790	2030–147,500
	Lotaustralin	100	32,6200 ± 34,640	16,850	32.1–115,900
	Total ^4^	100	74,800 ± 79,000	36,400	32.1–263,400
	Sum of plant metabolites	100	95,760 ± 81,560	85,700	32.1–265,3200
Unspecific	3-Nitropropionic acid	11	4.87 ± 1.91	4.87	3.52–6.22
	Brevianamid F	100	18.9 ± 13.7	14.1	6.50–62.4
	Citreorosein	50	18.1 ± 12.4	16.6	4.52–44.9
	cyclo(L-Pro-L-Tyr)	100	498 ± 347	361	172–1383
	cyclo(L-Pro-L-Val)	100	2190 ± 1000	1970	1080–4290
	Endocrocin	11	17.4 ± 6.77	17.4	12.6–22.1
	Iso-Rhodoptilometrin	22	2.25 ± 0.95	1.96	1.49–3.60
	Rugulusovine	100	13.7 ± 8.60	11.7	3.75–39.0
	Tryptophol	100	127 ± 118	74.0	53.1–485
	Sum of unspecific metabolites	100	2860 ± 1380	2460	1370–5910
	Sum of all detected metabolites	100	100,200 ± 80,900	92,100	4560–266,700

^1^ n = 18 pastures, samples with values > limit of detection (LOD); ^2^ Excluding data < LOD. In case values > LOD and <limit of quantification (LOQ), LOQ/2 was used for calculation; ^3^ emerging mycotoxins [37,38,39], ^4^ accumulative values of occurrences and concentrations of all the metabolites belonging to the group, ^5^ regulated mycotoxins (European Commission, 2002, 2006, 2012) [18,19,20] and ^6^ modified mycotoxins [21].

**Table 2:** Effect of the sampling season on the number of detected metabolites per sample and concentrations of the groups of metabolites.

Variable	Early	Late	SEM ^1^	*p*-Value
Number metabolites/sample				
All metabolites	24.4	39.6	3.51	0.008
Fungal metabolites	11.8	24.0	3.03	0.012
Concentration (µg/kg)				
from *Alternaria*	76	329	85.0	0.052
from *Aspergillus*	1.61	1.18	0.77	0.693
Ergot Alkaloids	5.32	110	44.6	0.120
from *Fusarium*	526	1890	431.8	0.041
from Lichen	1.76	1.56	0.81	0.865
from other fungi species	1.24	14.6	4.23	0.041
from *Penicillium*	0.00	0.76	0.50	0.303
Fungal Metabolites	611	2332	452	0.017
Phytoestrogens	7867	31,420	11,195	0.158
Cyanogenic glycosides	71,666	77,318	27,251	0.886
Plant metabolites	79,532	108,738	27,678	0.468
Unspecific metabolites	3144	4291	646	0.083
Total Metabolites	82,556	114,294	27,363	0.426

^1^ Values are least-squares mean (LS means) and SEM is the standard error of the LS means; Sampling season: Early = samples in April–June; Late = samples in August–October.

## Data Availability

Data available on request due to restrictions.

## Supplementary Materials

The following are available online at https://www.mdpi.com/article/10.3390/toxins13070460/s1, Table S1: List of 481 targeted metabolites via LC-MS/MS analysis. Compounds found in the pasture samples (values > the LOD) are located into grey cells, Table S2: Botanical composition of the sampled pastures.
