# Supplementary material for: Mycotoxins, Phytoestrogens and Other Secondary Metabolites in Austrian Pastures: Occurrences, Contamination Levels and Implications of Geo-Climatic Factors
Source: Toxins (Basel). 2021 Jun 30;13(7):460. doi: 10.3390/toxins13070460 (PMC8310091; doi:10.3390/toxins13070460)
Supplement: Supplementary file 1 [file toxins-13-00460-s001.zip › toxins-1255158-supplementary.pdf]

Supplementary Materials: Mycotoxins, Phytoestrogens, and Other Secondary Metabolites in Austrian Pastures: Occurrences, Contamination Levels, and Implications of Geo-climatic Factors

Felipe Penagos-Tabares, Ratchaneewan Khiaosa-ard, Veronika Nagl, Johannes Faas, Timothy Jenkins, Michael Sulyok and Qendrim Zebeli

Table S1. List of 481 targeted metabolites via LC-MS/MS analysis. Compounds found in the pasture samples (values > the LOD) are located into grey cells.

15-Acetyldeoxynivalenol	Aspyrone	Cladosporin	Emindole SA
15-Hydroxyculmorin	Asterric acid	Clonostachydiol	Emodin
15-Hydroxyculmoron	Atlantinon A	Colchicin	Endocrocin
2-Methylmitorubin	Atpenin A5	Communesin B	Enniatin A
3-Acetyldeoxynivalenol	Atropine	Cordycepin	Enniatin A1
3-Hydroxyterphenyllin	Atroventinmethylether	Coumestrol	Enniatin B
3-Nitropropionic acid	Aurantiamin A	Culmorin	Enniatin B1
4-Hydroxyalternariol	Aurantine	Curvularin	Enniatin B2
5-Hydroxyculmorin	Aurantioclavin	Curvulin	Enniatin B3
5-Methoxysterigmatocystin	Aurasperon B	cyclo(L-Leu-L-Pro)	Epiequisetin
5-Methylmellein	Aurasperon C	cyclo(L-Pro-L-Tyr)	Epoxyagroclavin
5-O-Methylsulochrin	Aurasperon G	cyclo(L-Pro-L-Val)	Epoxycytochalasin C
7-Hydroxykaurenolide	Aurofusarin	Cycloaspeptide A	Equisetin
7-Hydroxypestalotin	Austinol	Cycloheximide	Eremofortin A
Abscisic acid	Averantin	Cyclopenin	Eremofortin B
Acuminatum B	Averantinmethylether	Cyclopenol	Ergine
Acuminatum C	Averufanin	Cyclopeptine	Ergocornine
Aflatoxicol	Averufin	Cyclopiazonic acid	Ergocorninin
Aflatoxin B1	Bacitracin	Cylindrocarpon A4	Ergocristine
Aflatoxin B2	Bafilomycin A1	Cylindrol B	Ergocristinine
Aflatoxin G1	Barceloneic acid	Cytochalasin B	Ergocryptine
Aflatoxin G2	Bassianolide	Cytochalasin C	Ergocryptinine
Aflatoxin M1	Beauvericin	Cytochalasin D	Ergometrine
Aflatrem	Berkedrimane B	Cytochalasin E	Ergometrinine
Agroclavine	beta-Zearalenol	Cytochalasin H	Ergosin
Alamethicin	Bikaverin	Cytochalasin J	Ergosinin
alpha-Zearalenol	Biochanin	Daidzein	Ergotamine
Alteichin	Bis(methylthio)gliotoxin	Daidzin	Ergotaminine
Altenuene	BisdethioMTG	Deacetylneosolaniol	Ergovalin
Altenusin	Brefeldin A	Decalonectrin	Erucifolin
Alternariol	Brevianamid F	Dechlorogriseofulvin	Europin
Alternariolmethylether	Brevicompanine B	Dechloronornidulin	Europin-N-Oxid
Altersetin	Butenolid	Dehydroaustinol	Fallacinol
Altersolanol	Butyrolacton III	Dehydrocurvularin	Fellutanine A
Altertoxin-I	Butyrolactone I	Dehydrocyclopeptine	Festuclavine
Amauromine	Calonectrin	Demethylsulochrin	Flavipucin
Aminodimethyloctadecanol	Calphostin	Deoxyfusapyron	Flavoglaucin
Amoxycillin	Calyxanthone	Deoxynivalenol	Fonsecin
Amygdalin	Carviolin	Deoxynortryptoquivalin	Formonetin
Anacin	Cephalochromin	Deoxytryptoquivaline A	Fulvic acid
Andrastin A	Cercosporamide	Desoxyverrucosidin	Fumagillin
Andrastin B	Cercosporin	Destruxin A	Fumagillol
Andrastin C	Cereulide	Destruxin B	Fumifungin
Andrastin D	Chaconin	Destruxin-Ed Derivat	Fumigaclavine
Andrastin Derivative	Chaetominine	Diacetoxyscirpenol	Fumigaclavine C

Anisodamine	Chaetoviridin A	Dichlordiaportin	Fumiquinazolin A
Antibiotic F 1849 A	Chanoclavin	Dihydrocitrinone	Fumiquinazolin D
Antibiotic PF 1052	Chevalone B	Dihydroergosine	Fumiquinazolin Derivat
Antibiotic Y	Chevalone C	Dihydrogriseofulvin	Fumiquinazolin F
Apicidin	Chlamydospordiol	Dihydrolysergol	Fumitremorgin C
Apicidin D2	Chlamydosporol	Dihydrosterigmatocystin	Fumonisin A1
Ascochlorin	Chloramphenicol	Dihydroxycalonectrin	Fumonisin A1 Vorstufe
Ascofuranone	Chlorocitreorosein	Dihydroxymellein	Fumonisin A2
Aspercolorin	Chloronectrin	DihydroxyZONMethylether	Fumonisin B1
Asperfuran	Chlortetracyclin	Dinactin	Fumonisin B2
Asperglaucide	Chrodrimanin	Diplodiatoxin	Fumonisin B3
Asperlactone	Chrysogin	DON-3-glucoside	Fumonisin B4
Asperloxine A	Chrysophanol	Doxycyclin	Fumonisin B6
Asperphenamate	Citreohybridinol	Echimidin	Fungerin
Aspinolid B	Citreorosein	Elymoclavine	Fusaproliferin
Aspochracin A	Citreoviridin	Emericellamide A	Fusapyron
Aspterric acid	Citreoviridin C	Emericellamide C	Fusarenon-X
Aspulvinone E	Citrinin	Emericellamide E	Fusaric acid
Fusarin C	Methylequisetin	Prelaptin	Ternatin
Fusarinolic acid	Methylfunicone	Prunasin	Terphenyllin
Fuscofusarin	Methylorsellinic acid	Pseurotin A	Terrein
Geldanamycin	Methylsulochrin	Puromycin	Territrem B
Genistein	Mevinolin	Purpactin A	Tetracycline
Genistin	Mollicellin D	Purpuride	Thailandolide B
Gibberellic acid	Monactin	Pyranonigrin	Tiamulin
Gibberellin A14	Moniliformin	Pyrenocin A	Trichodermin
Gibepyron D	Monoacetoxyscirpenol	Pyrenophorol	Trichotetronine
Gliotoxin	Monocerin	Pyripyropene A	Trichothecolone
Glyantrypine	Monocrotalin	Pyripyropene D	Trypacidin
Glycitein	Mycophenolic acid	Pyrophen	Tryprostatin A
Glycitin	Mycophenolic acid IV	Quadrone	Tryprostatin B
Griseofulvin	Myriocin	Questiomycin A	Tryptophol
Griseophenone B	N-Benzoyl-Phenylalanine	Quinadoline A	Tryptoquivaline A
Griseophenone C	Neocyclocitrinol	Quinolactacin A	Tryptoquivaline F
Harzianopyridine	Neoechinulin D	Quinolactacin B	Tylosin
Heliotrin	Neosolaniol	Quinolone A	Unugisin E
Heliotrin-N-Oxid	Neoxaline	Radicicol	Usnic acid
Helvolic acid	NG 012	Rasfonin	Valinomycin
Helvolinic acid	Nidurufin	Roquefortine C	Vermistatin
Heptelidic acid	Nigericin	Roquefortine D	Verrucofortine
Herquline A	Nigragillin	Roridin A	Verrucosidin
HT-2 Glucoside	Nivalenol	Rubellin D	Verruculotoxin
HT-2 toxin	Nivalenol glucoside	Rubrofusarin	Versicolorin A
Hydroxycurvularin	Nonactin	Rugulosin	Versicolorin C
Hydroxypaspaline	Norlichexanthone	Rugulovasine A	Violaceic acid
Hydroxysydonic acid	Norsolorinic acid	Rugulusovin	Violaceol I
Hyoscin	Norverrucosidin	Sambucinol	Violaceol II
Ilicicolin A	Notoamide Derivative	Scalusamid A	Viomellein
Ilicicolin B	Ochratoxin A	Sclerotinin A	Viridicatin
Ilicicolin E	Ochratoxin alpha	Sclerotioramin	Viridicatol
Ilicicolin F	Ochratoxin B	Sclerotiorin	Viridicatum toxin
Indicin/Intermedin/Lycopsamin	Ochratoxin C	Secalonic acid D	Xanthomegnin
Indicin/Intermedin/Lycopsamin N-Oxid	O-Methylsterigmatocystin	Secalonic acid F	Xanthotoxin
Infectopyron	O-Methylviridicatin	seco-Sterigmatocystin	Zearalenone
Integracin A	Ononin	Senecionin_Senecivernin	Zearalenone-Sulfate
Integracin B	Orsellinic acid	Senkirkin	Zinndiol
Isofusidienol	Oxaline	Siccanin	Zinniamide
Iso-Rhodoptilometrin	Oxyskyrin	Siccanol	Zinniol
Jacobin	Oxytetracyclin	Sissotrine	

Josamycin	Papyracillic acid A	Skyrin
K-76 Derivative 4	Paraherquamide E	Solanin
Kojic acid	Paspalic acid	Sphingofungin B
Koninginin E	Paspalin	Sphingofungin D
Kotanin A	Paspalitrem A	Stachybotryamide
Lasiocarpin	Patulin	Stachybotrylactam
Lasiocarpin-N-Oxid	Paxillin	Staurosporin
Lecanoic acid	Penicillic acid	Stemphylperylenol
Linamarin	Penicillide	Sterigmatocystin
Lincomycin	Penicolinate	Sulochrin
LL-Z 1272e	Penigequinolone A	Surfactin A
Lolitrem B	Penitrem A	Surfactin B
Lotaustralin	Pestalotin	Sydonic acid
Macrosporin	Phenopyrrozin	Sydowinin A
Malformin A	Phomalactone	Sydowinin B
Malformin A2	Phomalone	T-2 Glucoside
Malformin C	Phomopsolide B	T-2 toxin
Marcfortine A	Physcion	T2-Tetraol
Marcfortine B	Pinselin	T2-Triol
Marcfortine C	Porritoxinol	Tensidol B
Meleagrin	Prehelminthosporol	Tentoxin
Methoxysterigmatocystin	Prehelminthosporollacton	Tenuazonic acid

Table S2. Botanical composition of the sampled pastures.

Sample	State	Botanical Composition (Dominant Species)[1]				
		1	2	3	4	5
1	LA	*Lolium perenne*	-	-	-	-
2	LA	*Lolium perenne*	*Trifolium repens*	*Dactylis glomerata*	-	-
3	LA	*Lolium perenne*	*Trifolium repens*	*Poa pratensis*	-	-
4	LA	*Phleum pratense*	*Trifolium repens*	-	-	-
5	LA	*Lolium perenne*	*Trifolium repens*	-	-	-
6	UA	*Lolium perenne*	*Trifolium repens*	*Medicago sativa*	-	-
7	UA	*Lolium perenne*	*Medicago sativa*	-	-	-
8	UA	*Lolium perenne*	*Trifolium pratense*	*Dactylis glomerata*	-	-
9	UA	*Lolium perenne*	*Trifolium repens*	-	-	-
10	UA	*Lolium perenne*	*Trifolium repens*	-	-	-
11	ST	*Lolium perenne*	*Alopecurus pratensis*	*Holcus lanatus*	*Poa pratensis*	-
12	ST	*Poa pratensis*	*Alopecurus pratensis*	*Festuca pratensis*	-	-
13	ST	*Dactylis glomerata*	*Phleum pratense*	*Trifolium repens*	*Cynosurus cristatus*	*Festuca pratensis*
14	ST	*Lolium perenne*	*Trifolium repens*	*Dactylis glomerata*	*Agrostis sp.*	-
15	ST	*Dactylis glomerata*	*Trifolium repens*	-	-	-
16	ST	*Lolium perenne*	*Medicago sativa*	*Buglossoides sp.*	*Cynosurus cristatus*	*Dactylis glomerata*
17	ST	*Lolium perenne*	*Trifolium repens*	-	-	-
18	ST	*Dactylis glomerata*	*Trifolium repens*	-	-	-

LA: Lower Austria; UP: Upper Austria; ST: Styria; [1] Taxonomical classification based on morphological keys.
